# Structural Engineering in Sn-Doped WO_3_ Multi-Phase Systems for Enhanced Transparent Heat Insulation

**DOI:** 10.3390/molecules30204124

**Published:** 2025-10-17

**Authors:** Xinyu Song, Ze Wang, Yue Liu, Xin Li, Chun Du, Shifeng Wang

**Affiliations:** Key Laboratory of Plateau Oxygen and Living Environment of Xizang Autonomous Region, College of Science, Xizang University, Lhasa 850000, China; songxinyu@stu.utibet.edu.cn (X.S.); wangze@stu.utibet.edu.cn (Z.W.); liuyue@stu.utibet.edu.cn (Y.L.); lixin@stu.utibet.edu.cn (X.L.)

**Keywords:** building energy conservation, transparent thermal insulation, WO_3_, structural regulation

## Abstract

Building energy conservation through the development of transparent thermal insulation materials that selectively block near-infrared radiation while maintaining visible light transmittance has emerged as a key strategy for global carbon neutrality. WO_3_ is a semiconductor oxide with near-infrared absorption capabilities. However, the limited absorption efficiency and narrow spectral coverage of pure WO_3_ significantly diminish its overall transparent thermal insulation performance, thereby restricting its practical application in energy-saving glass. Therefore, this study successfully prepared Sn-doped WO_3_ materials using a one-step hydrothermal method, controlling the Sn:W molar ratio from 0.1:1 to 2.0:1. Through evaluation of transparent thermal insulation performance of a series of Sn-doped WO_3_ samples, we found that Sn:W = 0.9:1 exhibited the most excellent performance, with NIR shielding efficiency reaching 93.9%, which was 1.84 times higher than pure WO_3_. Moreover, this sample demonstrated a transparent thermal insulation index (THI) of 4.38, representing increases of 184% and 317%, respectively, compared to pure WO_3_. These enhancements highlight the strong NIR absorption capability achieved by Sn-doped WO_3_ through structural regulation. When Sn doping reaches a certain concentration, it triggers a structural transformation of WO_3_ from monoclinic to tetragonal phase. After reaching the critical solubility threshold, phase separation occurs, forming a multiphase structure composed of a Sn-doped WO_3_ matrix and secondary SnO_2_ and WSn_0.33_O_3_ phases, which synergistically enhance oxygen vacancy formation and W^6+^ to W^5+^ reduction, achieving excellent NIR absorption through small polaron hopping and localized surface plasmon resonance effects. This study provides important insights for developing high-performance transparent thermal insulation materials for energy-efficient buildings.

## 1. Introduction

With social development and rapid urbanization processes, modern buildings have become increasingly energy-intensive, with building energy consumption accounting for approximately 40% of global energy consumption [1]. To maintain indoor thermal comfort, heating, ventilation, and air conditioning (HVAC) systems consume a substantial portion of building energy, particularly in cooling applications, which can account for up to 60% of total building energy consumption [2,3]. Solar radiation entering buildings through conventional glass windows represents the primary source of unwanted heat gain, significantly increasing cooling loads and energy costs. Solar energy primarily consists of three components, ultraviolet (UV), visible light (Vis), and near-infrared radiation (NIR), with NIR radiation accounting for approximately 50% of total solar energy and serving as the primary source of thermal energy [4,5,6]. Conventional glass materials exhibit 80–90% transmittance in both visible and near-infrared wavelength ranges, lacking the spectral selectivity required for effective thermal management [7,8]. Therefore, developing spectrally selective materials that can effectively shield NIR radiation while maintaining high visible light transmittance is of paramount importance for reducing building energy consumption and achieving carbon emission reduction targets.

Numerous approaches have been investigated to address challenges in building thermal management, among which transparent thermal insulation materials represent a promising solution that can be broadly categorized into two types: passive thermal barrier systems such as vacuum glazing, and transparent thermal insulation coatings applied to conventional glass that can selectively absorb or reflect specific wavelength bands [9]. Vacuum glazing systems consist of two glass sheets with a vacuum space between them, utilizing conduction and convection mechanisms to reduce heat transfer [10]. The primary manufacturing challenges encountered by vacuum glazing include maintaining vacuum conditions, addressing edge sealing issues, and achieving large-scale production, which have hindered its widespread application [11,12]. In comparison, transparent thermal insulation coatings offer more practical implementation feasibility using conventional coating processes and can provide effective control over solar radiation [13,14,15]. Among various coating materials, tungsten-based oxides have attracted attention due to their unique optoelectronic properties and NIR absorption characteristics [16].

Tungsten trioxide (WO_3_) and its derivative materials have emerged as research hotspots in the transparent thermal insulation field owing to their unique electronic structures and optical properties [17,18]. Although WO_3_ exhibits certain NIR absorption capabilities, its optical performance can be significantly enhanced through various modification strategies, including structural regulation, compositional control, and surface modification [19,20,21]. Among compositional control approaches, incorporating other ions into WO_3_, such as transition metal ions (Fe^3+^, Co^3+^, etc.) and non-metal ions (N^3−^, F^−^, etc.), has been found to alter the electronic structure and band structure of WO_3_, thereby affecting its optical properties [22,23]. Recent studies have explored various dopants with different performance characteristics, among which alkali metal doping (particularly Cs) exhibits strong near-infrared absorption through localized surface plasmon resonance but faces challenges of high cost and moisture sensitivity [24]; while rare earth element doping (such as La and Ce) can improve material stability, it is limited by restricted doping concentrations and modest enhancement in NIR absorption [25]. However, most doping strategies encounter limitations including restricted near-infrared shielding wavelength ranges, insufficient stability, and raw material cost constraints [26,27]. Tin (Sn) doping offers unique advantages for WO_3_ modification due to the favorable ionic radius compatibility between Sn^4+^ (0.69 Å) and W^6+^ (0.60 Å), with both Sn and W typically having coordination numbers of 6, as they are coordinated to six oxygen atoms in the WO_3_ structure [28]. Furthermore, the charge difference between Sn^4+^ and W^6+^ provides driving force for defect formation, subsequently enhancing near-infrared shielding efficiency through synergistic effects of localized small polaron transitions and localized surface plasmon resonance (LSPR) [29,30]. However, despite these theoretical advantages, certain issues in Sn-doped WO_3_ systems remain unresolved. The solubility limit of Sn in the WO_3_ lattice under hydrothermal conditions has not been quantitatively determined. The structural changes beyond this threshold, particularly the occurrence of phase separation and the formation of secondary phases, remain unclear. More critically, the mechanistic correlation between these structural transitions, the resulting defect chemistry (oxygen vacancies and W^5+^ centers), and the enhancement of near-infrared shielding performance has not been systematically established. These issues hinder the rational design and optimization of Sn-doped WO_3_ materials for practical transparent thermal insulation applications.

To address these critical questions, we systematically synthesized Sn-doped WO_3_ materials via hydrothermal method with Sn:W molar ratios controlled from 0.1:1 to 2.0:1. The hydrothermal method was selected due to its unique advantages, including low-temperature synthesis, excellent control over particle morphology and dopant distribution, and cost-effectiveness for large-scale production [31]. Results demonstrated that upon doping to a certain level, WO_3_ underwent transformation from monoclinic to tetragonal phase, accompanied by approximately 50% reduction in unit cell volume. When the doping concentration exceeded a certain threshold, phase separation occurred, forming a multiphase structure composed of a Sn-doped WO_3_ matrix and secondary SnO_2_ and WSn_0.33_O_3_ phases. Optical performance evaluation revealed that the Sn:W = 0.9:1 molar ratio achieved the most excellent transparent thermal insulation effect, exhibiting 93.9% NIR shielding efficiency and a transparent thermal insulation index of 4.38, representing increases of 184% and 317%, respectively, compared to pure WO_3_. The enhanced performance originated from structural changes induced by Sn doping, which promoted oxygen vacancy formation and created favorable conditions for efficient NIR absorption. This study provides effective strategies for he performance optimization of transparent thermal insulation materials and offers guiding value for the development of building tenergy-saving technologies.

## 2. Results and Discussion

**Figure 1:**
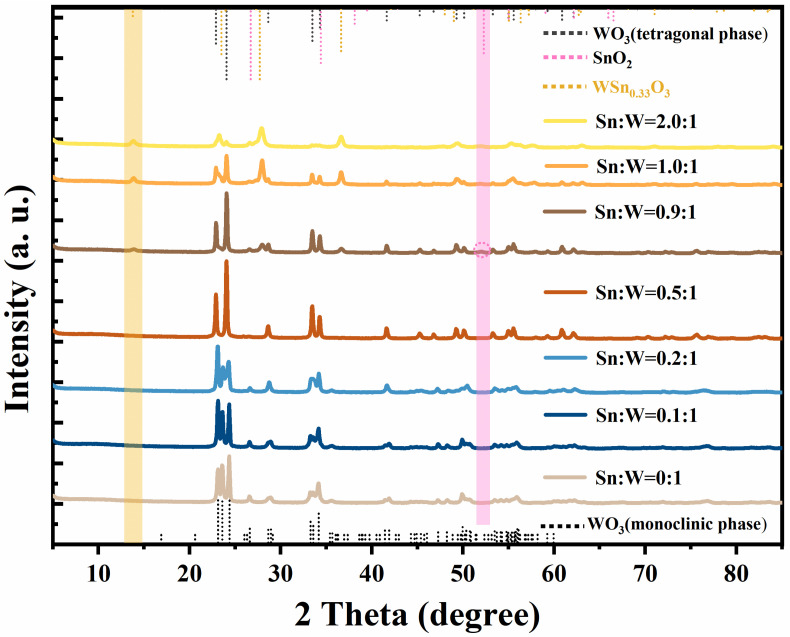
XRD patterns of samples with different Sn:W molar ratios (0:1, 0.1:1, 0.2:1, 0.5:1, 0.9:1, 1.0:1, and 2.0:1).

**Figure 2:**
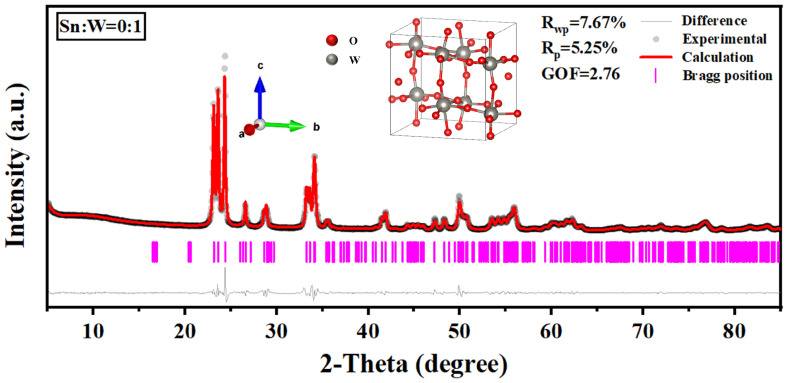
Rietveld refinement plot of Sn:W = 0:1.

**Figure 3:**
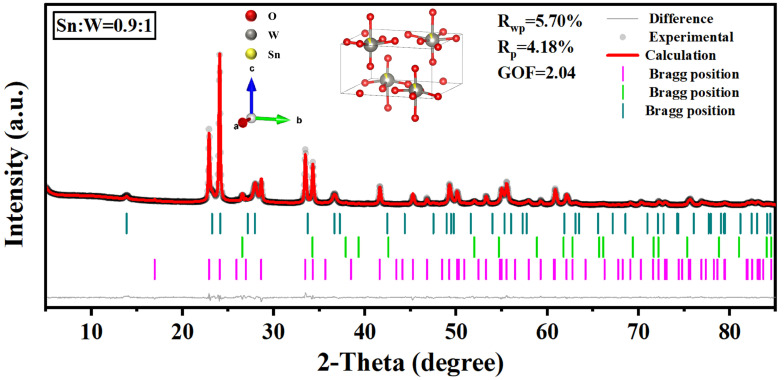
Rietveld refinement plot of Sn:W = 0.9:1 sample, with an inset showing the crystal structure of WO_3_.

Figure 1 shows the X-ray diffraction (XRD) spectra analysis of pure WO_3_ and Sn-doped WO_3_ samples. From Figure 1, it can be observed that at low doping levels (Sn:W = 0:1, 0.1:1, 0.2:1), all samples’ XRD spectra show excellent matching with the standard card of WO_3_ (PDF#00-043-1035), confirming the successful preparation of WO_3_. These samples maintain the characteristics of the monoclinic crystal system with the space group P 2_1_/n, and lattice parameters of a = 7.303 Å, b = 7.536 Å, c = 7.693 Å, α = γ = 90°, β = 90.91°. In addition, the characteristic diffraction peaks observed at 23.1°, 23.6°, 24.3°, 33.3°, and 34.2° correspond to the (002), (020), (200), (022), and (220) planes of monoclinic WO_3_, respectively. The sharpness and high intensity of these peaks provide strong evidence that the prepared material has excellent crystallinity and phase purity. However, when the doping concentration increases to Sn:W = 0.5:1, a fundamental change in the crystal structure of the system occurs. The XRD analysis clearly reveals the formation of a complex multiphase system, with the most notable feature being the structural reconstruction of the WO_3_ main phase from monoclinic to tetragonal. This crystallographic transition results in a change of space group to P $\bar{4}$2_1_m (PDF#97-008-6144), accompanied by a re-adjustment of lattice parameters: a = b = 7.39 Å, c = 3.88 Å, α = β = γ = 90°. Correspondingly, the characteristic diffraction peaks observed at 22.9°, 24.1°, 28.6°, 33.5°, and 34.3° correspond to the (001), (200), (111), (201), and (220) planes of tetragonal WO_3_ phase, marking the reorganization of the main phase structure. More importantly, distinct secondary phase signals also appear in the XRD spectra: diffraction peaks at 13.9° and 28.0° correspond to the (100) and (200) planes of hexagonal WSn_0.33_O_3_ (PDF#04-010-3202), while the strong peak at 52.0° corresponds to the (211) plane of tetragonal SnO_2_ (PDF#97-016-9032). This multiphase coexistence phenomenon confirms the successful doping of Sn. The Rietveld refinement results presented in Figure 2 and Figure 3, combined with the quantitative data in Table 1 and Table 2, provide detailed evidence for a deeper understanding of the mechanism of this complex structural change. From the refinement quality in Figure 2, it can be seen that the refinement parameters are R_wp_ = 7.67%, R_p_ = 5.25%, and GOF = 2.76. The high degree of agreement between the experimental and theoretical calculated spectra, along with the minimal difference curve, confirms the perfect single-phase characteristics and the high reliability of the structural model. In sharp contrast, Figure 3 shows that the refinement parameters are R_wp_ = 5.70%, R_p_ = 4.18%, and GOF = 2.04. The refinement results for the Sn:W = 0.9:1 sample indicate that phase separation occurred in the sample. The crystal structure illustration shows that Sn atoms, represented by yellow spheres, occupy the W sites within the tetragonal lattice framework, confirming the substitutional doping of Sn. At the same time, the Bragg peak positions confirm the coexistence of a three-phase system, consisting of Sn-doped WO_3_ phase, SnO_2_ phase, and WSn_0.33_O_3_ phase. Quantitative data in Table 1 show that the pure WO_3_ sample maintains 100% phase purity and a stable monoclinic structure (cell volume = 423.378 Å^3^), while the Sn:W = 0.9:1 sample exhibits a complex three-phase coexistence state: 75.7 wt% Sn-doped WO_3_ main phase (tetragonal, volume = 212.110 Å^3^), 4.5 wt% SnO_2_ secondary phase (volume = 70.559 Å^3^), and 19.8 wt% WSn_0.33_O_3_ ternary phase (hexagonal, volume = 179.857 Å^3^). Notably, the volume of the WO_3_ phase decreases sharply from 423.378 Å^3^ to 212.110 Å^3^, representing about a 50% volume compression. This volume change reflects the structural reconstruction that occurs during the monoclinic to tetragonal phase transition, with the main mechanisms involving compression along the c-axis and an increase in crystal symmetry [32]. Table 2 further reveals the structural changes induced by Sn doping. First, from the perspective of structural symmetry, the transition from monoclinic to tetragonal represents an enhancement of the space group symmetry. Secondly, the sharp reduction in the cell volume from 423.378 Å^3^ to 212.110 Å^3^, a 50% compression, reflects a fundamental reorganization of the three-dimensional atomic arrangement: systematic shortening of atomic distances, redistribution of bond angles, and an increase in overall lattice density. An even more crucial finding is the transition from a single-phase to a multiphase system, which provides direct evidence for determining the solubility limit of Sn in WO_3_. Although the set stoichiometric ratio during synthesis was Sn:W = 0.9:1, the Rietveld refinement results show that the actual Sn occupancy in the WO_3_ main phase is only 8.8 at.%, which clearly defines the thermodynamic solubility limit of Sn in the WO_3_ lattice under hydrothermal conditions. When the introduced Sn content exceeds this critical threshold, the material will form two phases, SnO_2_ and WSn_0.33_O_3_, to effectively release the oversaturated Sn atoms.

**Figure 4:**
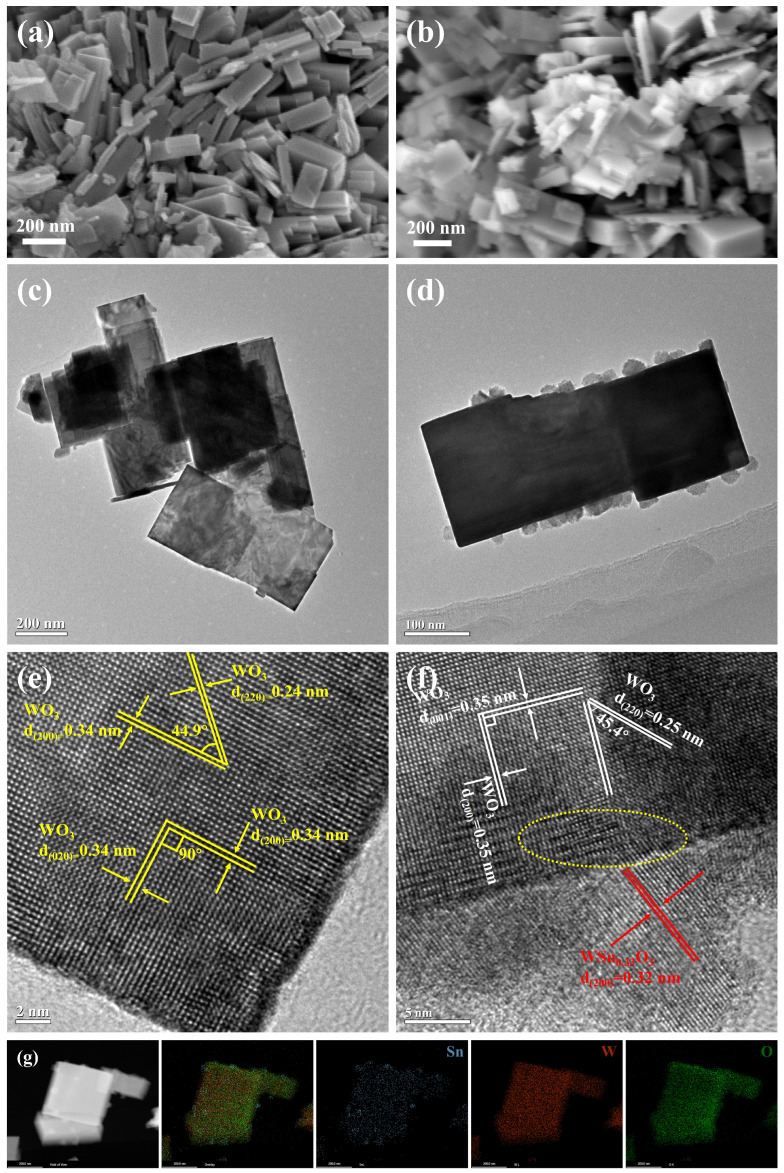
SEM and TEM images of Sn:W = 0:1 and Sn:W = 0.9:1 samples: (**a**,**c**,**e**) Sn:W = 0:1; (**b**,**d**,**f**,**g**) Sn:W = 0.9:1.

Figure 4 presents the morphological and microstructural characterization results of Sn:W = 0:1 and Sn:W = 0.9:1 samples. From the scanning electron microscope images in Figure 4a,b, it is clear that Sn doping significantly affects the morphology of the WO_3_ samples. The monoclinic WO_3_ sample shown in Figure 4a exhibits a relatively regular block-like morphology, with clear edge contours, a relatively dispersed spatial distribution, and good particle size uniformity. However, after the introduction of Sn, as shown in Figure 4b, small granular substances appeared on the surface of the material, accompanied by some degree of aggregation. This morphological change is likely closely related to the complex reorganization process occurring during the transition of the crystal structure from monoclinic to tetragonal. To further analyze the microstructural characteristics, Figure 4c,d present the transmission electron microscope (TEM) images of Sn:W = 0:1 and Sn:W = 0.9:1 samples, respectively. Through higher-resolution TEM observations, it can be more clearly seen that Sn doping indeed leads to a significant change in the morphology of the sample, especially with the surface-grown granular structures becoming more clearly visible. By measuring the interplanar distances in Figure 4e, a 0.24 nm interplanar spacing corresponds to the (220) plane of monoclinic WO_3_, while a 0.34 nm interplanar spacing found at a 44.9° angle corresponds to the (200) plane of WO_3_, and this angle shows an excellent match with the theoretical value of 44.1°. Additionally, another 0.34 nm interplanar spacing found in the 90° direction to the (220) plane corresponds to the (020) plane, with the angle relation perfectly matching the theoretical value of 90°. The systematic analysis of these interplanar distances and angle relations further conclusively proves that the phase generated in the Sn:W = 0:1 sample is indeed the standard monoclinic WO_3_ phase. In contrast, the HRTEM analysis of the Sn-doped sample in Figure 4f reveals a more complex multiphase structural feature. By measuring the interplanar distances, the characteristic interplanar distance of the tetragonal WO_3_ (220) plane can be identified, while the interplanar spacing of the (200) plane found at a 45.4° angle is very close to the theoretical value of 45°. Further, another 0.35 nm interplanar spacing in the 90° direction to the (200) plane corresponds to the (001) plane, and its angle relation perfectly matches the theoretical value of 90%, providing solid crystallographic evidence for the successful preparation of tetragonal WO_3_. More importantly, within the same field of view, a 0.32 nm interplanar spacing corresponding to the (200) plane of WSn_0.33_O_3_ phase was also found, perfectly corroborating the crystal structural features revealed in the previous XRD analysis. Moreover, at the phase boundary, we also observed the presence of lattice defects, which manifested as noticeable lattice distortion and bending, in stark contrast to the highly regular lattice structure surrounding them. The formation of these local defect structures is mainly attributed to the significant size mismatch effect caused by Sn^4+^ ions (ionic radius 0.69 Å) replacing W^6+^ ions (ionic radius 0.60 Å). Figure 4g shows the TEM-EDS element distribution mapping results for the Sn:W = 0.9:1 sample. From the element distribution map, it can be observed that Sn is relatively uniformly distributed in the particles, with no apparent local enrichment or aggregation. Meanwhile, W and O elements remain uniformly distributed across the entire testing area.

**Figure 5:**
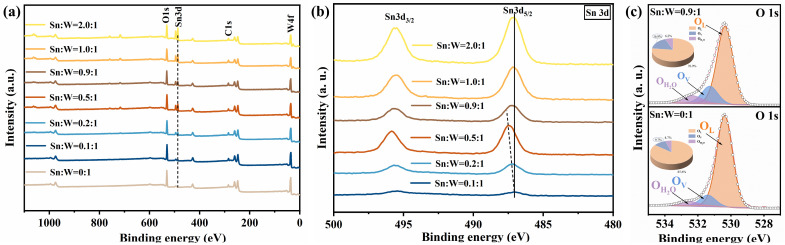
XPS spectra of WO_3_ and samples with different Sn doping levels: (**a**) full spectrum, (**b**) Sn 3d, and (**c**) O 1s.

Figure 5 presents the X-ray photoelectron spectroscopy (XPS) results of WO_3_ and samples with different Sn doping levels. From the full XPS spectrum in Figure 5a, it can be observed that the pure WO_3_ sample (Sn:W = 0:1) primarily shows characteristic peaks for W 4f, O 1s, and C 1s. As the Sn:W molar ratio gradually increases from 0.1:1 to 2.0:1, the intensity of the Sn 3d characteristic peaks shows a noticeable increasing trend, further confirming the successful incorporation of Sn. The high-resolution Sn 3d spectrum analysis in Figure 5b shows that as the Sn doping concentration systematically increases, the Sn 3d_5/2_ peak exhibits a chemical shift behavior, first shifting negatively and then positively. This non-monotonic binding energy change reflects the dynamic variation of the chemical environment around the Sn atoms. At low doping concentrations, the negative shift of the Sn 3d_5/2_ peak may be attributed to Sn^4+^ ions substituting W^6+^ sites; while the positive shift observed at higher doping concentrations is likely related to the different chemical environments created by the Sn-rich phase formed during phase separation, which corresponds well with the multiphase coexistence observed in the previous XRD analysis. The O 1s spectrum in Figure 5c was fitted and analyzed, revealing that the O 1s spectrum can be decomposed into three distinct components, corresponding to lattice oxygen (O_L_), oxygen vacancies (O_V_), and adsorbed water molecules (O_H2O_)^29^. Comparative analysis shows that for the Sn:W = 0.9:1 sample, the fitted area of the oxygen vacancy (O_V_) component increased compared to the pure WO_3_ sample (Sn:W = 0:1), indicating that the Sn doping process increased the oxygen vacancy defects.

**Figure 6:**
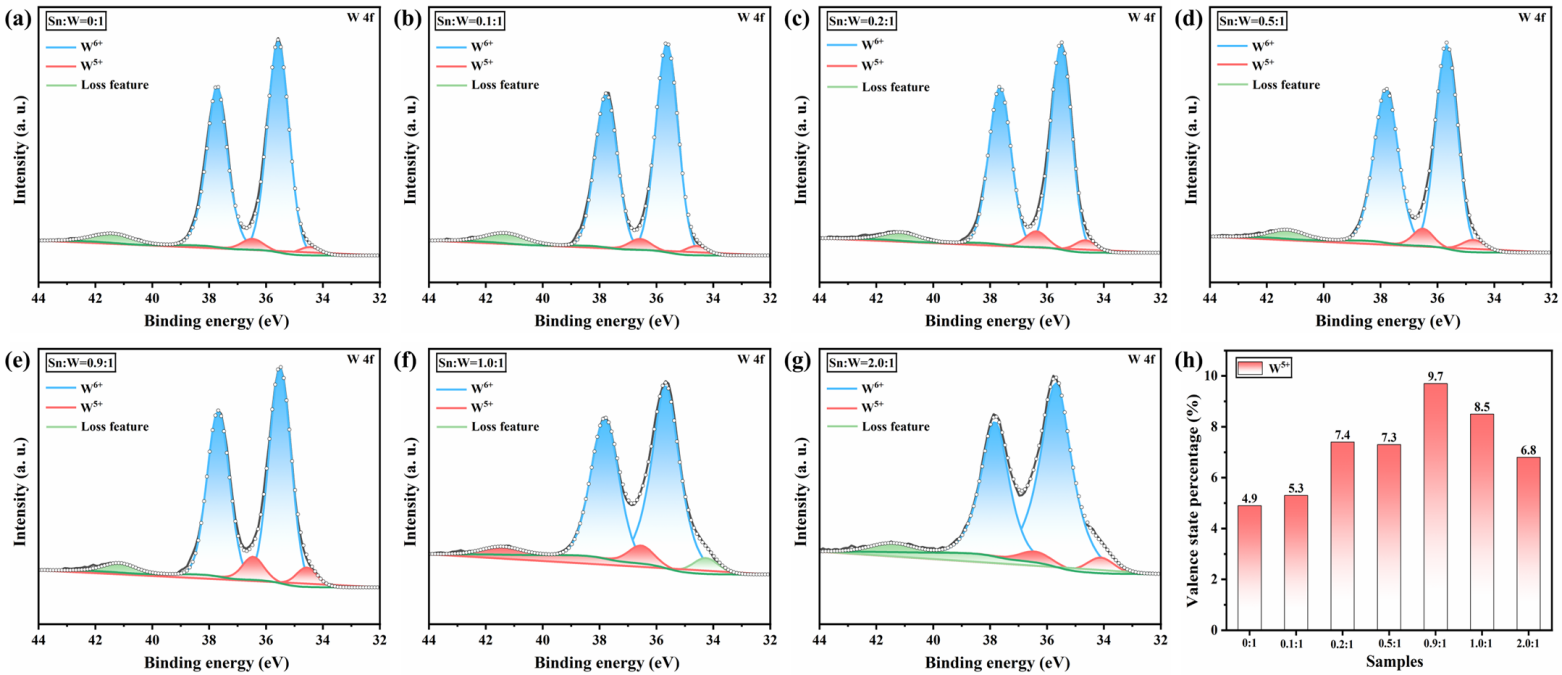
High-resolution W 4f XPS spectra of samples with different Sn:W molar ratios: (**a**) Sn:W = 0:1, (**b**) Sn:W = 0.1:1, (**c**) Sn:W = 0.2:1, (**d**) Sn:W = 0.5:1, (**e**) Sn:W = 0.9:1, (**f**) Sn:W = 1.0:1, (**g**) Sn:W = 2.0:1, and (**h**) quantitative analysis of the percentage of W^5+^ states as a function of Sn doping concentration.

Figure 6a–h show the W 4f XPS spectra of samples with different Sn:W molar ratios. The W 4f spectrum can be decomposed into two main parts, representing the W^5+^ and W^6+^ oxidation states, with characteristic peaks located at 34.5 eV/36.5 eV and 35.6 eV/37.7 eV, respectively. The quantitative analysis in Figure 6h provides the proportion of W^5+^ species in the six studied materials. A trend can be observed from Figure 6h, where the proportion of W^5+^ increases with the increase in the Sn:W ratio, reaching a maximum value at Sn:W = 0.9:1. This finding suggests that Sn doping promotes the conversion of more W^6+^ ions to W^5+^ ions. The significance of this valence state transformation lies in that the W^5+^ centers and their associated oxygen vacancy defects provide key electronic states for NIR optical reactions. These defect levels act as localized centers for charge carriers, facilitating the formation of small polarons. This leads to broad, continuous infrared absorption bands due to carrier transitions between these localized states. Additionally, these defects induce localized surface plasmon resonance (LSPR) modes, which enhance optical shielding in the near-infrared range by efficiently scattering infrared light [30,33,34].

**Figure 7:**
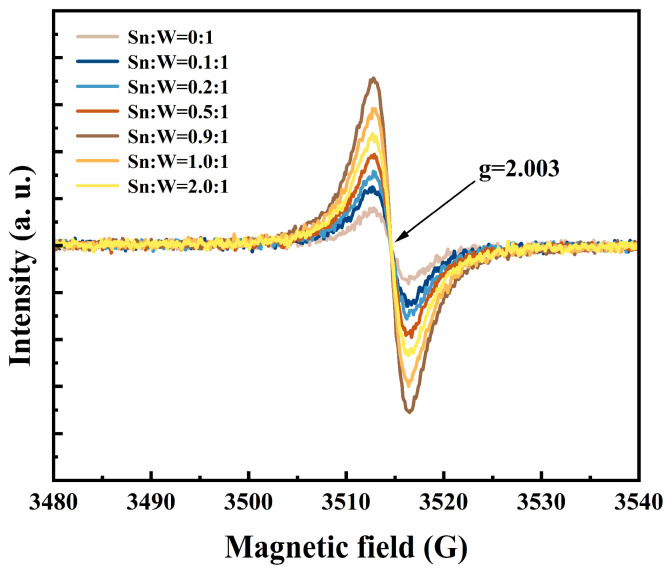
Electron paramagnetic resonance (EPR) spectra of samples with different Sn:W molar ratios.

Figure 7 shows the EPR spectra of samples with different Sn:W molar ratios. The EPR signal at g = 2.003 comes from unpaired electrons at the oxygen vacancy sites and can be used to characterize the oxygen vacancy density. EPR data show that the oxygen vacancy concentration in all Sn-doped samples is higher than in pure WO_3_, and it exhibits a trend of first increasing and then decreasing with the increase in Sn content, reaching a maximum value at Sn:W = 0.9:1. This trend is consistent with the change in the W^5+^ proportion observed in the XPS analysis. The increase in oxygen vacancy concentration will have a certain impact on the electronic and optical properties of the material. From an electronic perspective, oxygen vacancies introduce additional defect states within the bandgap, which act as localized centers for charge carriers, increasing the carrier concentration and enhancing the mobility of charge carriers by providing alternative pathways for electron transport. This leads to an increase in the carrier transport rate. More importantly, oxygen vacancies promote the formation of W^5+^ centers, and this defect-mediated valence state regulation is beneficial for near-infrared absorption [35,36].

**Figure 8:**
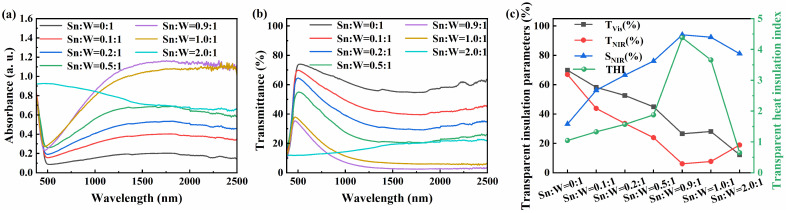
(**a**) Optical absorption spectra of samples with different Sn:W molar ratios; (**b**) transmittance spectra calculated from absorption data; (**c**) comparison of transparent thermal insulation performance parameters of samples with different Sn:W molar ratios.

Figure 8 shows the optical properties and transparent thermal insulation performance of samples with different Sn:W molar ratios. Figure 8a presents the UV-Vis-NIR absorption spectra in the wavelength range of 380–2500 nm, Figure 8b shows the corresponding transmission spectra calculated from the absorption data (Equation (1)), while Figure 8c includes a comprehensive evaluation of transparent thermal insulation parameters. The analysis of Figure 8a shows that pure WO_3_ (Sn:W = 0:1) exhibits minimal absorption across all spectral ranges, particularly in the near-infrared region. Upon Sn doping, the absorption intensity of the material increases significantly, reaching a maximum at Sn:W = 0.9:1, especially in the near-infrared range (780–2500 nm) that is critical for thermal insulation applications. Correspondingly, as shown in Figure 8b, the Sn:W = 0.9:1 sample possesses optimal wavelength selectivity with the lowest transmittance in the near-infrared wavelength range. Figure 8c uses quantitative optical parameter analysis to provide an objective numerical evaluation of transparent thermal insulation performance. Visible light transmittance (T_Vis_), near-infrared transmittance (T_NIR_), near-infrared shielding rate (S_NIR_), and transparent thermal insulation index (THI) are calculated using established formulas. The transmittance (T) is calculated from the absorbance (A) using the relationship:

T = 10^−A^ (1)

where higher absorbance corresponds to lower transmittance. The evaluation parameters are defined as follows [37]:

(2) $$T_{\mathrm{Vis}}\;=\;\frac{\int_{380}^{780}T\;\left(\lambda \right)\;d\;\left(\lambda \right)}{780\;-\;380}\;\times \;100\%$$

(3) $$T_{\mathrm{NIR}}=\frac{\int_{780}^{2500}T\;\left(\lambda \right)\;d\;\left(\lambda \right)}{2500-780}\;\times \;100\%$$

S_NIR_ = 100% − T_NIR_ (4)

**Scheme 1:**
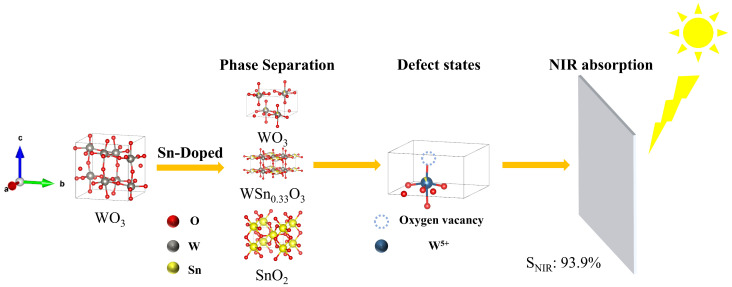
Mechanistic pathway of enhanced near-infrared absorption in Sn-doped WO_3_ transparent thermal insulation materials.

THI = T_Vis_/T_NIR_ (5)

where T (λ) is the transmittance spectrum within the selected wavelength range. The results show that the Sn:W = 0.9:1 sample exhibits the best transparent thermal insulation performance. Its near-infrared shielding rate (S_NIR_) reaches 93.9%, an increase of 60.8 percentage points compared to the pure WO_3_ sample (Sn:W = 0:1) at 33.1%, representing an improvement of 184%. This fully demonstrates the significant role of Sn doping in enhancing infrared absorption. Meanwhile, the transparent thermal insulation index (THI) reaches 4.38, an increase of 3.17 times compared to the 1.05 of pure WO_3_, indicating that this component achieves an optimal balance between visible light transmittance and near-infrared shielding. Table 3 summarizes the optical properties of the Sn–W samples, including the transmittance at 550 nm (T@550 nm), the average visible transmittance (T_Vis_) in the 380–780 nm range, and the average near-infrared transmittance (T_NIR_) in the 780–2500 nm range. In addition, the table provides the solar near-infrared shielding efficiency (S_NIR_) and the thermal insulation index (THI). As seen in Table 3, with increasing Sn doping, the visible transmittance (T_Vis_) decreases while the near-infrared transmittance (T_NIR_) and S_NIR_ increase, which suggests that higher Sn content enhances the thermal insulation performance of the samples. To elucidate the underlying mechanism responsible for this superior performance, we present a comprehensive structure-property relationship in Scheme 1.

**Figure 9:**
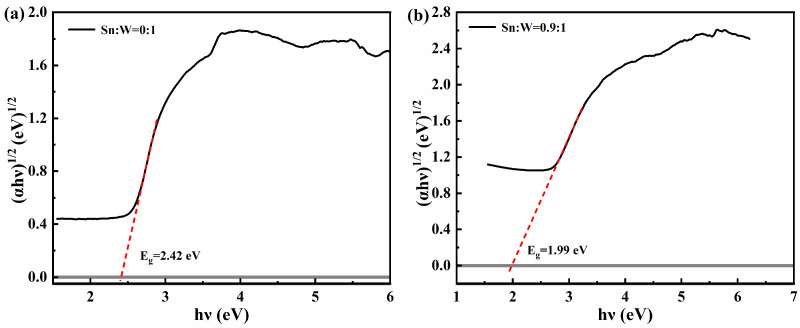
Tauc plots of Sn–W samples: (**a**) Sn:W = 0:1 (pure WO_3_); (**b**) Sn:W = 0.9:1.

To investigate the effect of Sn doping on the band gap of Sn–W samples, Tauc plots were constructed based on absorption spectra. As shown in Figure 9, the Tauc plot of the pure WO_3_ sample (Sn:W = 0:1) reveals an indirect band gap of 2.42 eV. For the Sn-doped sample (Sn:W = 0.9:1), the band gap decreases to 1.99 eV, indicating that Sn doping induces band gap narrowing. Since both transparency and near-infrared (NIR) absorption are influenced by the absorption characteristics of the material, the reduction in the band gap is directly related to changes in transparency and NIR absorption. As the band gap narrows, the material exhibits enhanced absorption of near-infrared light, thereby improving near-infrared shielding efficiency and the transparent thermal insulation index. Conversely, the narrowing of the band gap also results in a decrease in visible light transmittance, as evidenced by the reduction in the Tvis value.

To further highlight the advantages of the Sn–W samples, we compared their near-infrared shielding efficiency (S_NIR_) with that of other reported materials. As shown in Table 4, the Sn:W = 0.9:1 sample in this study exhibits an SNIR of 93.9%, which is superior to that of other materials.

## 3. Materials and Methods

### 3.1. Materials

The chemical reagents used in this study were applied directly without further purification. Sodium tungstate (Na_2_WO_4_·2H_2_O, AR, 99.5%) and oxalic acid dihydrate (C_2_H_2_O_4_·2H_2_O, GR, 99.8%) were purchased from Shanghai Macklin Biochemical Technology Co., Ltd. (Shanghai, China) Anhydrous stannous chloride (SnCl_2_, 99%) was purchased from Shanghai Aladdin Biochemical Technology Co., Ltd. (Shanghai, China) Hydrochloric acid (HCl, AR, 36–38%) was provided by Chengdu Kolon Chemicals Co., Ltd. (Chengdu, China) Anhydrous ethanol (C_2_H_5_OH, AR, ≥99.7%) was purchased from Tianjin Zhiyuan Chemical Reagent Co., Ltd. (Tianjin, China). Ultrapure water (resistivity 18.25 MΩ cm^−1^) was prepared using the ULUPURE water purification system (model UPR-II-10TNZ) and was used throughout all experiments.

### 3.2. Preparation of WO_3_

Dissolve sodium tungstate (Na_2_WO_4_·2H_2_O, 0.5 g, 1.51 mmol) and oxalic acid dihydrate (C_2_H_2_O_4_·2H_2_O, 0.3 g, 2.38 mmol) in 100 mL deionized water, then slowly add 4 mL hydrochloric acid (HCl), and stir with a magnetic stirrer for 2 h to obtain a uniform solution. Pour the solution into a 100 mL polytetrafluoroethylene (PTFE, Shanghai Xiniu Leibo Instrument Co., Ltd., Shanghai, China) liner and carry out hydrothermal reaction at 180 °C for 18 h. After allowing the solution to naturally cool to room temperature, collect the precipitate by centrifugation and wash it alternately with water and ethanol three times. Finally, dry the washed material in a vacuum oven (Shanghai Jinghong Experimental Equipment Co., Ltd., Shanghai, China) at 60 °C for 12 h to obtain pure WO_3_ material.

### 3.3. Synthesis of Sn-Doped WO_3_ (SnWO_3_)

SnWO_3_ composite materials were synthesized using a similar hydrothermal method, with the synthesis procedure largely identical to that for WO_3_, the main difference being the addition of anhydrous stannous chloride (SnCl_2_) during the preparation of the initial solution. Specifically, predetermined amounts of SnCl_2_ (0.028, 0.058, 0.144, 0.259, 0.287, and 0.575 g) were dissolved in 100 mL deionized water along with Na_2_WO_4_·2H_2_O (0.5 g, 1.51 mmol) and C_2_H_2_O_4_·2H_2_O (0.3 g, 2.38 mmol) to obtain precise Sn:W molar ratios of 0.1:1, 0.2:1, 0.5:1, 0.9:1, 1.0:1, and 2.0:1. The resulting samples were named Sn:W = 0.1:1, Sn:W = 0.2:1, Sn:W = 0.5:1, Sn:W = 0.9:1, Sn:W = 1.0:1, and Sn:W = 2.0:1. All subsequent steps were carried out following the procedure used for the synthesis of pure WO_3_. Undoped WO_3_ samples (Sn:W = 0:1) were used as control references for comparison and analysis.

### 3.4. Characterizations

The crystal structure and phase composition of the synthesized samples were characterized using a high-resolution X-ray diffractometer (TD-3700 model, Dandong Tongda Technology Co., Ltd., Dandong, China), with CuKα radiation (λ = 1.5406 Å), and Rietveld refinement analysis was performed using the General Structure Analysis System (GSAS-II ver.5782) software package to determine lattice parameters and crystallographic information. The morphology and microstructure of the samples were observed using a scanning electron microscope (SEM5000 model, CIQTEK Co., Ltd., Hefei, China), and high-resolution transmission electron microscope (HRTEM) images and elemental distribution maps were obtained using a field emission transmission electron microscope (Tecnai G2 F20 model, FEI, Hillsboro, OR, USA). X-ray photoelectron spectroscopy (XPS) was performed using an X-ray photoelectron spectrometer (Escalab 250XI model, ThermoFisher, Waltham, MA, USA), with monochromatic AlKα radiation (hν = 1486.6 eV) and X-ray power of 150 W. The test parameters were as follows: analysis area diameter of 650 µm, acceleration voltage of 14.8 kV, and emission current of 1.6 A. All binding energies were calibrated using the contaminant carbon C1s peak (284.8 eV) as a reference. EPR spectra were recorded using a Bruker A300-10/12 electron paramagnetic resonance (EPR) spectrometer (Bruker, Billerica, MA, USA). The optical properties, transparency, and near-infrared shielding performance of the samples were evaluated using an ultraviolet-visible-near infrared spectrophotometer (UV-3600 model, Shimadzu Corporation, Kyoto, Japan), with a testing wavelength range of 200–2500 nm.

### 3.5. Optical Characterization (Diffuse Reflectance Measurement)

Barium sulfate is filled into the integrating sphere, and the background peak is measured first. Approximately 20 mg of the sample is then taken, evenly spread and pressed onto the surface of the barium sulfate, and the UV spectrum is measured using diffuse reflectance. The measurement range is from 200 to 2500 nm, with a step size set to 1 nm.

## 4. Conclusions

This study successfully synthesized Sn-doped WO_3_ materials with excellent transparent thermal insulation properties via hydrothermal method and investigated the mechanism of enhanced near-infrared absorption. Structural characterization revealed that when the doping level exceeded Sn:W = 0.5:1, Sn incorporation induced a crystallographic transformation from monoclinic to tetragonal WO_3_, establishing the thermodynamic solubility limit of Sn under hydrothermal conditions at 8.8 at.%. XPS and EPR analyses demonstrated that Sn doping facilitated the reduction of W^6+^ to W^5+^ and oxygen vacancy formation through a charge compensation mechanism. The W^5+^ content reached a maximum of 9.7% at Sn:W = 0.9:1, which coincided with the peak oxygen vacancy concentration. This electronic modification achieved enhanced near-infrared absorption through small polaron hopping and localized surface plasmon resonance effects. Optical performance evaluation showed that the Sn:W = 0.9:1 sample exhibited optimal transparent thermal insulation properties, with near-infrared shielding efficiency (S_NIR_) reaching 93.9% (184% improvement compared to pure WO_3_) and transparent thermal insulation index (THI) of 4.38 (3.17 times higher than pure WO_3_). The enhanced NIR absorption originated from structural regulation induced by Sn doping, where Sn^4+^ substitution for W^6+^ triggered monoclinic to tetragonal phase transformation reconstruction, forming a multiphase coexistence system when exceeding the 8.8 at.% solubility limit, with phase transformation and multiphase interfaces synergistically promoting oxygen vacancy formation and subsequently achieving efficient near-infrared light absorption. This study provides design guidance for the structural optimization of doped tungsten oxides and offers an effective pathway for the development of energy-efficient building materials.

## Figures and Tables

**Table 1:** Comparison of the Rietveld refinement results of Sn:W = 0:1 and Sn:W = 0.9:1.

Sample	Phase Composition	Space Group	Unit Cell Parameters (Å)
Sn:W = 0:1	100% WO_3_	P 2_1_/n (monoclinic)	a = 7.3031, b = 7.5360, c = 7.6934
Sn:W = 0.9:1	75.7% WO_3_ (Sn-doped)	P $\bar{4}$2_1_m (tetragonal)	a = b = 7.3937, c = 3.8800
Sn:W = 0.9:1	4.5% SnO_2_	P 4_2_/mnm (tetragonal)	a = b = 4.7441, c = 3.1350
Sn:W = 0.9:1	19.8% WSn_0.33_O_3_	P 6/mmm (hexagonal)	a = b = 7.3681, c = 3.8254

**Table 2:** Structural changes induced by Sn doping.

Parameter	Sn:W = 0:1	Sn:W = 0.9:1
Crystal System	Monoclinic	Tetragonal (main phase)
Unit Cell Volume	423.378 Å^3^	212.110 Å^3^ (main phase)
Phase Purity	Single phase	Multi-phase system
Sn Occupancy	0%	8.8 at.% in WO_3_ phase

**Table 3:** Summary of optical properties (T@550 nm, Tvis, TNIR, SNIR, THI) of Sn–W samples.

Sample	T@550 nm	T_Vis_, Avg (380–780 nm)	T_NIR_, Avg (780–2500 nm)	S_NIR_	THI
Sn:W = 0:1	88.7%	69.9%	66.9%	33.1%	1.05
Sn:W = 0.1:1	68.4%	58.2%	43.8%	56.2%	1.32
Sn:W = 0.2:1	62.6%	52.6%	33.5%	66.5%	1.57
Sn:W = 0.5:1	53.9%	44.9%	23.9%	76.1%	1.88
Sn:W = 0.9:1	32.2%	26.6%	6.1%	93.9%	4.38
Sn:W = 1.0:1	33.5%	28.1%	7.7%	92.3%	3.66
Sn:W = 2.0:1	11.9%	12.3%	18.9%	81.1%	0.65

**Table 4:** Comparison of near-infrared shielding efficiency (S_NIR_) of Sn–W samples with other materials: PDLC, CWO@ATO-10, and Sn:W = 0.9:1 from this work.

Sample	SNIR	Reference
PDLC	80%	[38]
CWO@ATO-10	93.1%	[39]
Sn:W = 0.9:1	93.9%	This work

## Data Availability

Data are contained within the article.
